# Orbital Exenteration for Craniofacial Lesions: A Systematic Review and Meta-Analysis of Patient Characteristics and Survival Outcomes

**DOI:** 10.3390/cancers15174285

**Published:** 2023-08-27

**Authors:** Jumanah Qedair, Ali S. Haider, Kishore Balasubramanian, Paolo Palmisciano, Taimur Hassan, Ataollah Shahbandi, Mohammadmahdi Sabahi, Abdurrahman F. Kharbat, Hussam Abou-Al-Shaar, Kenny Yu, Aaron A. Cohen-Gadol, Tarek Y. El Ahmadieh, Othman Bin-Alamer

**Affiliations:** 1 College of Medicine, King Saud bin Abdulaziz University for Health Sciences, Jeddah 22384, Saudi Arabia; qedair042@ksau-hs.edu.sa; 2 King Abdullah International Medical Research Center (KAIMRC), Jeddah 22384, Saudi Arabia; 3 Department of Neurosurgery, The University of Texas MD Anderson Cancer Center, Houston, TX 77030, USA; 4 Texas A&M School of Medicine, Texas A&M University, Houston, TX 77030, USA; 5 Department of Neurological Surgery, University of California, Davis, Sacramento, CA 95819, USA; 6 Tehran School of Medicine, Tehran University of Medical Science, Tehran 1416634793, Iran; 7 Department of Neurological Surgery, Pauline Braathen Neurological Centre, Cleveland Clinic Florida, Weston, FL 33331, USA; 8 Department of Neurosurgery, The University of Oklahoma, Oklahoma City, OK 73019, USA; 9 Department of Neurosurgery, University of Pittsburgh Medical Center, Pittsburgh, PA 15219, USA; 10 Department of Neurosurgery, Memorial Sloan Kettering Cancer Center, New York, NY 10065, USA; 11 Department of Neurological Surgery, Indiana University School of Medicine, Indianapolis, IN 46202, USA; 12 Department of Neurosurgery, Loma Linda University, Loma Linda, CA 92354, USA

**Keywords:** orbital exenteration, craniofacial, carcinoma, survival, outcomes, systematic review, meta-analysis

## Abstract

**Simple Summary:**

Notwithstanding its disfiguring nature, orbital exenteration (OE) has been employed as a surgical intervention for craniofacial lesions, especially in cases of advanced or recurrent tumors. There is a dearth of large studies investigating the clinical and survival outcomes of this procedure. Our study aims to review the literature on the clinical characteristics and outcomes of patients who underwent OE. In the univariable analysis, we found that a positive surgical margin after OE was significantly associated with worse overall survival (OS). Conversely, sex, tumor recurrence, type of OE, and tumor histopathology were not found to exert significant effects on OS.

**Abstract:**

Background: The outcomes of orbital exenteration (OE) in patients with craniofacial lesions (CFLs) remain unclear. The present review summarizes the available literature on the clinical outcomes of OE, including surgical outcomes and overall survival (OS). Methods: Relevant articles were retrieved from Medline, Scopus, and Cochrane according to PRISMA guidelines. A systematic review and meta-analysis were conducted on the clinical characteristics, management, and outcomes. Results: A total of 33 articles containing 957 patients who underwent OE for CFLs were included (weighted mean age: 64.3 years [95% CI: 59.9–68.7]; 58.3% were male). The most common lesion was squamous cell carcinoma (31.8%), and the most common symptom was disturbed vision/reduced visual acuity (22.5%). Of the patients, 302 (31.6%) had total OE, 248 (26.0%) had extended OE, and 87 (9.0%) had subtotal OE. Free flaps (33.3%), endosseous implants (22.8%), and split-thickness skin grafts (17.2%) were the most used reconstructive methods. Sino-orbital or sino-nasal fistula (22.6%), flap or graft failure (16.9%), and hyperostosis (13%) were the most reported complications. Regarding tumor recurrences, 38.6% were local, 32.3% were distant, and 6.7% were regional. The perineural invasion rate was 17.4%, while the lymphovascular invasion rate was 5.0%. Over a weighted mean follow-up period of 23.6 months (95% CI: 13.8–33.4), a weighted overall mortality rate of 39% (95% CI: 28–50%) was observed. The 5-year OS rate was 50% (median: 61 months [95% CI: 46–83]). The OS multivariable analysis did not show any significant findings. Conclusions: Although OE is a disfiguring procedure with devastating outcomes, it is a viable option for carefully selected patients with advanced CFLs. A patient-tailored approach based on tumor pathology, extension, and overall patient condition is warranted.

## 1. Introduction

Orbital exenteration (OE) is an invasive surgical procedure intended to remove the orbit contents in cases of lesions invading the orbital cavity and periocular structures [1,2,3]. Since its description in the 16th century by Bartisch [4,5], it has been performed for the surgical management of a variety of conditions, including malignancies, infections, trauma, and inflammatory diseases [3,6,7,8]. The most common indications of OE are craniofacial cancers, including squamous cell carcinoma (SCC) and basal cell carcinoma (BCC) [2,8,9,10]. OE can result in a wide range of complications, most commonly sino-orbital fistulas, graft or flap failure, and surgical site infection or non-healing wounds [4,10,11,12,13,14,15].

Different surgical techniques for OE have emerged over the past decades, including ablative surgery, total OE, subtotal or eyelid-sparing OE, and extended OE [16]. While ablative surgery focuses on eliminating tumor tissue with negative margins, total OE removes all orbit content, subtotal OE spares some orbital tissue, and extended OE removes adjacent structures beyond the orbit. Additional surgeries, such as lymph node dissection, may be performed simultaneously [1,16,17,18]. Following OE, several reconstructive techniques are usually considered to cover the evacuated orbit and surrounding region as needed. These techniques vary from spontaneous granulation to skin grafts, regional flaps, and free flaps [6,8,9,19]. In addition, cosmetic rehabilitation, in the form of a prosthesis or other socket-reconstructive methods, may be discussed with patients to ameliorate the damaging impact of OE on their quality of life, including self-image and social functioning [4,20,21,22,23].

In this study, we aimed to address the scattered data on the clinical and survival outcomes of patients with craniofacial lesions (CFLs) managed with OE. The existing literature primarily consists of case reports and series. By conducting a comprehensive systematic review of the literature, we sought to provide a comprehensive summary of the findings and bridge the knowledge gaps regarding the role of OE in the management of CFLs, as well as its associated clinical and survival outcomes.

## 2. Materials and Methods

### 2.1. Literature Search

A systematic review and meta-analysis, registered with PROSPERO (ID: CRD42023429608), were conducted in accordance with the Preferred Reporting Items for Systematic Reviews and Meta-Analyses (PRISMA) guidelines [24]. The databases of Medline, Scopus, and Cochrane were comprehensively searched from their inception until July 2022. A search strategy, employing both medical subject headings (MeSH) and terms and keywords, was implemented using the Boolean operators OR and AND. The search terms utilized were “Orbital OR Orbit” AND “Exenteration.” The retrieved papers were imported into Mendeley (Version 2.80.1, Mendeley Ltd., July 2022, London, UK), and any duplicate records were eliminated.

### 2.2. Study Selection

Inclusion and exclusion criteria were defined to ensure study selection consistency. Studies were included if they (1) were retrospective or prospective studies of adult patients (≥18 years) who underwent OE procedure for CFLs and (2) reported data on clinical features, procedural details, and treatment outcomes. Studies were excluded if they (1) were meta-analyses, reviews, editorials, letters, books, or case reports, (2) contained insufficient clinical data (i.e., lacking patient demographics or management details and outcomes), (3) included 5 or less patients, (4) presented inseparable data of pediatric and adult patients or inseparable data of OE and other procedures, or (5) were written in a foreign language.

The titles and abstracts of all extracted papers were independently evaluated by two authors (KB and JQ), using the predetermined inclusion and exclusion criteria. Then, studies that met the inclusion criteria underwent further independent assessment through a full-text review by the same two authors. In cases of discrepancies, a third author (OBA) was consulted to resolve them. Additionally, the references of the included articles were screened to identify any additional relevant studies.

### 2.3. Data Extraction

Data from the studies that met the inclusion criteria were extracted by one author (JQ) and independently verified by another author (OBA) to ensure accuracy. The extracted variables included the author’s name, publication date, level of evidence, sample size, sex, symptoms at presentation, histological and clinical characteristics, management approaches and treatment modalities, complications, recurrence rates, and survival outcomes. Missing data were either not reported by the authors or were indistinguishable from other data.

### 2.4. Data Synthesis and Quality Assessment

The primary outcomes of interest were the overall survival (OS) and survival predictive factors. The secondary objectives were summarizing the characteristics of the CFLs (e.g., tumor histopathology) that required OE procedure, the management course including the type and extent of OE, and local and distant recurrence. The OS was reported according to the follow-up and survival protocol of the original papers. The level of evidence of each article was assessed using the 2011 Oxford Centre for Evidence-Based Medicine guidelines, and all articles were classified as level IV evidence [25]. Two authors (JQ and ASH) independently evaluated the risk of bias for each article using the Joanna Briggs Institute checklists [26]. The assessment revealed that all the included papers had a low risk of bias (Supplementary Table S1). Throughout this article, we followed the extent of resection classification for OE as proposed by Frezzotti et al. [27]. They categorized OE as subtotal, total, and radical, ranging from I to VI. Subtotal OE contains types I, II, and III, all of which spare the eyelid skin. Type I spares palpebral and bulbar conjunctiva, type II spares palpebral conjunctiva only, and type III is limited to sparing the deeper muscle layer. The second category, total OE, is composed of type IV only, where the eyelid skin is resected. The third category, radical, also called extended OE, includes types V and VI. In type V, the orbit cavity bones are resected, and in type VI, the resection is extended to adjacent structures. Tumor recurrence was classified as local, regional, and distant, just as they were reported in the included articles.

### 2.5. Statistical Analysis

The statistical analyses in this study were conducted using Stata software (StataCorp. 2023. Stata Statistical Software: Release 17. College Station, TX, USA: StataCorp. LLC.). Continuous variables were summarized by reporting weighted means (effect size of means) and 95% confidence intervals (CIs). Categorical variables, on the other hand, were summarized by reporting frequencies and percentages or weighted proportions (effect size of proportions) and 95% CIs. Missing observations in any variables were excluded from the analysis. The survival data were presented as median survival in months, accompanied by 95% CIs, and 5-year and 10-year survival rates, which were visualized using Kaplan–Meier curves [28]. The log-rank test was employed to assess the null hypothesis of no difference in survival across the categories of each categorical variable. To examine potential factors influencing survival, both univariable and multivariable analyses were conducted using the Cox proportional hazards model [29]. Only variables that were statistically significant in the univariable analysis were included in the multivariable analysis. To assess the proportional hazards assumption, Schoenfeld’s global test was employed to estimate time-varying covariance in the multivariable analysis. The results indicated that the assumption was met. Two-tailed *p*-value < 0.05 was considered statistically significant for all analyses conducted. Data from all included studies were pooled, and a meta-analysis was performed using the random effects model. To summarize pooled proportions, a meta-analysis of proportion was conducted, while a meta-analysis of means was employed to summarize pooled means. Subgroup analysis was conducted to assess statistical differences among various groups. Heterogeneity was assessed using the χ^2^ test and the Higgins *I*^2^ test [30]. Publication bias was evaluated using funnel plots and Egger’s test, with a *p*-value less than 0.05 indicating the presence of bias [31]. We confirmed that all studies demonstrated visual symmetry, and none of the tested endpoints revealed publication bias (Supplementary Figure S1).

## 3. Results

### 3.1. Study Selection

**Figure 1:**
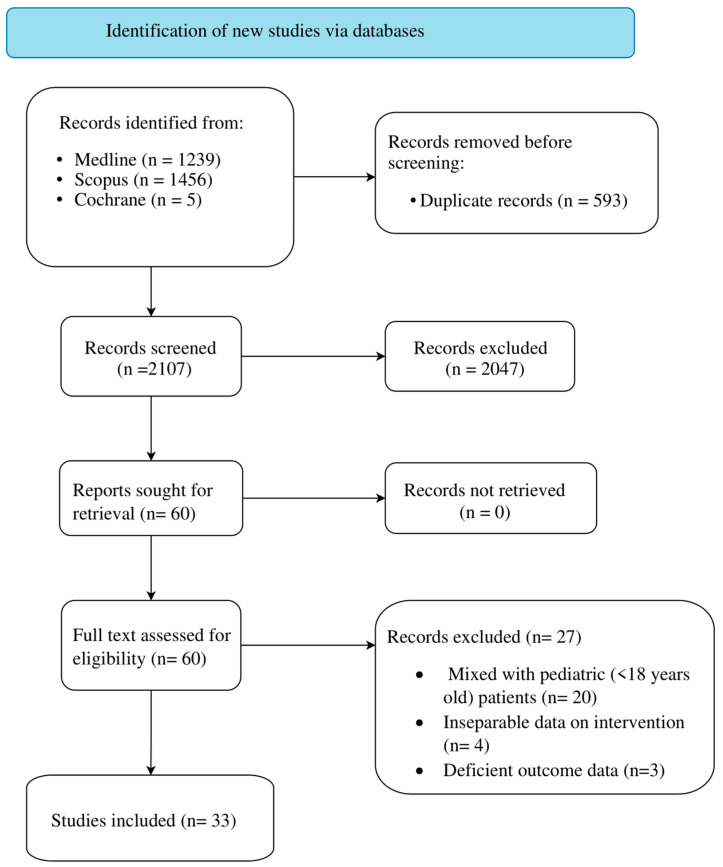
PRISMA 2020 Flow Diagram.

The initial literature search of databases yielded a total of 2700 articles (Figure 1). After removing duplicate records, the number of articles was reduced to 2107. Out of these, 2047 studies were excluded based on the screening of their titles and abstracts. Sixty papers were selected for retrieval and were assessed for inclusion through a full text review. Among the assessed articles, 27 did not meet our inclusion criteria and were subsequently excluded. Therefore, 33 articles, which were categorized as level IV evidence, were included in the analysis (Supplementary Table S2) [2,3,4,6,8,9,10,11,12,13,14,15,17,18,19,23,32,33,34,35,36,37,38,39,40,41,42,43,44,45,46,47,48].

### 3.2. Demographics and Clinical Characteristics of the Cohort

**Figure 2:**
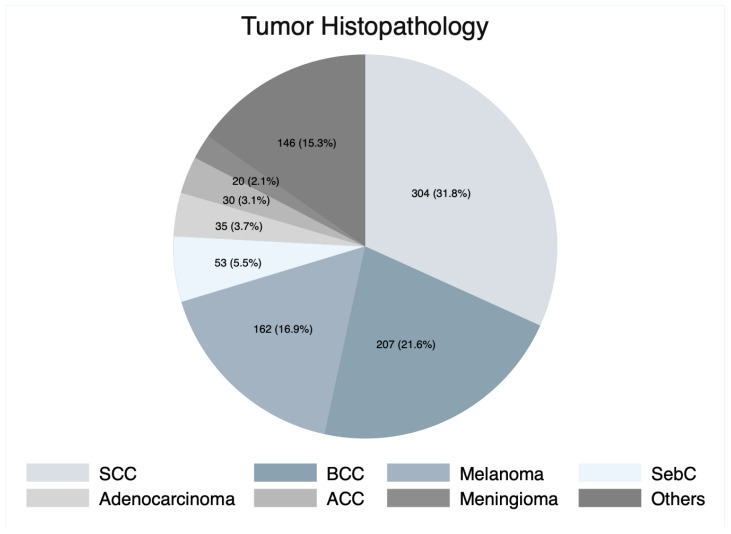
Pie chart of tumor histopathology. SCC, squamous cell carcinoma; BCC, basal cell carcinoma; SebC, sebaceous cell carcinoma; ACC, adenoid cystic carcinoma.

In our cohort, a total of 957 patients who underwent OE were included, among which 558 (58.3%) were male. The weighted mean patient age was 64.3 years (95% CI: 59.9–68.7 years). The most frequently involved anatomical structures were the eyelid (16.2%), orbit (14.4%), and conjunctiva (12.5%) (Table 1). SCC was the most common histopathological diagnosis (*n* = 304, 31.8%), followed by BCC (*n* = 207, 21.6%), and melanoma (*n* = 162, 16.9%) (Figure 2). The most common signs and symptoms at presentation were disturbed vision (reduced visual acuity) (22.5%), facial mass (17.7%), conjunctival injection (14.7%), eye pain (13.0%), and lesion discharge (12.0%) (Table 1).

### 3.3. Patient Management

Around one-third of the patients (*n* = 302, 31.6%) underwent total OE, and the other two-thirds underwent extended (*n* = 248, 26.0%), subtotal (*n* = 87, 9.0%), or unspecified (*n* = 320, 33.4%) OE (Table 2). The term “unspecified OE” is used to describe OE cases where the type and extent (total, subtotal, or extended) were not clearly specified in the original articles. A group of patients (*n* = 146) required additional surgical interventions, such as maxillectomy (39.0%), lymph node resection (24.7%), ethmoidectomy (11.6%), and craniotomy (9.6%). A total of 168 (17.6%) patients experienced complications after OE. The most common complications included sino-orbital or sino-nasal fistulas (22.6%), flap or graft failure (16.9%), hyperostosis (13.0%), wound dehiscence or non-healing (10.7%), and cerebrospinal fluid leak (8.5%) (Table 2).

Following OE, various reconstructive techniques were utilized (Table 3). The most commonly employed reconstructive techniques were free flap (33.3%), endosseous implants (22.8%), and split-thickness skin grafting (17.2%). The anterolateral thigh flap was the most used reconstructive material (26.6%), followed by the musculocutaneous flap (21.6%), and the radial forearm flap (14.5%).

Of the total 957 patients, 414 (43.3%) received one or more radiation therapy treatments. Specifically, 266 patients (62.7%) underwent adjuvant radiotherapy, while 106 (25.0%) and 52 (12.3%) underwent primary and neoadjuvant radiotherapies, respectively. Chemotherapy was used in 47 patients as neoadjuvant (*n* = 16, 31.4%) or adjuvant (*n* = 35, 68.0%) therapies. The study revealed that only 29 patients received both chemotherapy and radiotherapy before or after OE (Table 4).

### 3.4. Clinical and Survival Outcomes

Out of the 957 patients included in the review, data on tumor recurrence were available in 210 (22.0%) patients. Among these, 38.6% (*n* = 109) had local recurrence, while 32.3% (*n* = 91) and 6.7% (*n* = 19) experienced distant and regional recurrence, respectively. In addition, CFLs invaded perineural and lymphovascular regions in 17.4% and 5.0% of the patients, respectively. Notably, some patients experienced multiple intracranial and extracranial tumor recurrences (Table 5).

**Figure 3:**
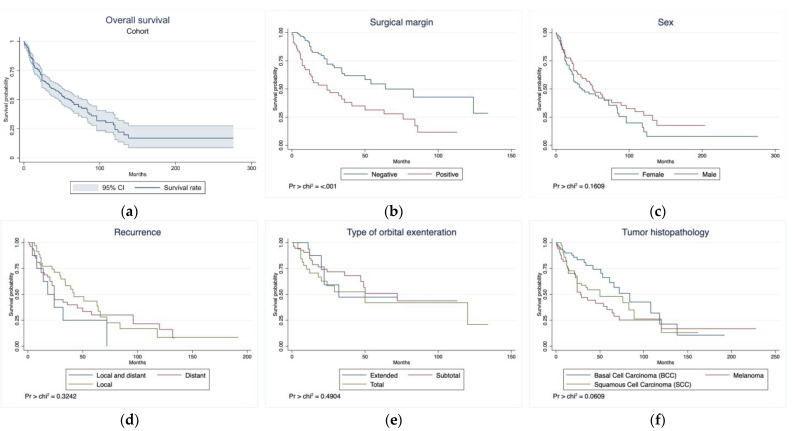
Kaplan–Meier curves of the (**a**) overall survival of the entire cohort and overall survival based on (**b**) surgical margins, (**c**) sex, (**d**) recurrence, (**e**) type of orbital exenteration, and (**f**) tumor histopathology. Pr > chi^2^ denotes the *p* value of log-rank test.

During a weighted mean follow-up time of 23.6 months (95% CI: 13.8–33.4 months), 215 (36.6%) patients died, and the cause of death was reported in only 195 patients. Among these, 144 (73.8%) patients died due to disease-related complications (Table 5). The 5-year OS rate was 50.0%, and the median OS time was 61 months (95% CI: 46–83 months; Table 5; Figure 3a). The presence of positive surgical margins was associated with a lower survival time (median: 24 months, 95% CI: 12–41 months) compared with negative surgical margins (median: 64 months, 95% CI: 36–NA months, *p* < 0.001; Figure 3b). Sex, tumor recurrence, type of the OE, and tumor histopathology did not show any statistically significant survival effect (Figure 3c–f).

Multivariable analysis of OS using the Cox proportional hazards model did not result in any significant survival predictors (Table 6).

**Figure 4:**
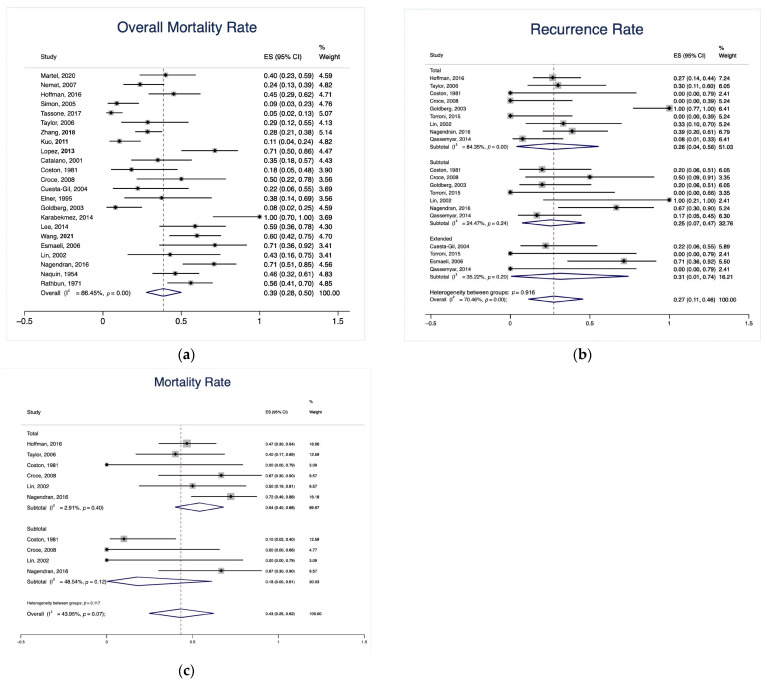
Forest plot of (**a**) overall mortality rate of the cohort, (**b**) tumor recurrence rate based on the type of OE, and (**c**) mortality rate based on the type of OE. OE, orbital exenteration [2,3,4,6,8,9,10,11,12,14,17,18,19,34,35,37,40,41,42,43,44,45,46,47,48].

The total cohort exhibited a weighted overall mortality rate of 39.0% (95% CI: 28.0–50.0%; Figure 4a). In the subgroup meta-analysis, the extended OE group had an insignificantly higher rate of tumor recurrence (31.0% [95% CI: 1.0–74.0%]) compared with the total OE group (26.0% [95% CI: 4.0–56.0%]) and the subtotal OE group (25.0% [95% CI: 7.0–47.0%], *p* = 0.92; Figure 4b). The total OE group had an insignificantly higher mortality rate (54.0% [95% CI: 40.0–68.0%]) compared with the subtotal OE group (18.0% [95% CI: 0.0–61.0%], *p* = 0.12; Figure 4c).

## 4. Discussion

OE serves as a surgical treatment for advanced or recurrent CFLs. While the existing literature covers OE extensively, comprehensive studies concerning survival outcomes post-procedure are sparse. Univariable analysis showed a correlation between positive surgical margins post-OE and diminished OS. Sex, tumor recurrence, type of OE, and tumor histopathology did not significantly affect OS.

### 4.1. Demographics and Clinical Characteristics of the Cohort 

We found that the majority of patients (58.3%) were male, with a weighted mean age of 64.3 years (95% CI: 59.9–68.7 years), consistent with other studies on CFLs [49,50,51,52]. OE was primarily indicated for lesions invading the orbit and adjacent structures. Thus, the eyelid (16.2%), orbit (14.4%), and conjunctiva (12.5%) were frequently affected in our study. SCC was the predominant histopathological indication for OE (31.8%), followed by BCC (21.6%), and melanoma (16.9%). Similar to our results, several other reports indicated a higher incidence of SCC affecting the craniofacial skull base structures [9,10,14,15,53].

Various symptoms were reported by patients who subsequently became eligible for OE. Among these, disturbed vision was the most prevalent symptom (22.5%) in the present study, which is reasonable given the anatomical location of CFLs [54].

### 4.2. Management Paradigm and Complications

We found that a third (31.6%) of patients underwent total OE, while 26.0% and 9.0% had extended OE and subtotal OE, respectively, demonstrating the variability in resection extents required by different patient conditions. The extent of resection directly correlates with disfigurement severity, impacting patients’ self-perception and life quality [55].

In the subgroup meta-analysis, our findings indicated that the types of OE, extended, total, and subtotal, for CFLs had no significant differences in terms of recurrence and mortality rates. These results are in line with other studies that have demonstrated no significant effect of the OE type on survival outcomes [56]. Further research should focus on identifying the optimal OE type to balance improved survival rates and the minimization of complications.

Multiple surgical procedures—such as maxillectomy (39.0%), lymph node resection (24.7%), ethmoidectomy (11.6%), and craniotomy (9.6%)—were necessary for some patients, underscoring the invasiveness of OE and CFLs. Similarly, many studies in the literature have evinced the need for adjunctive surgeries to eradicate lesions involving adjacent structures, particularly the lymph nodes, cranial and facial bones [1,57,58]. These findings highlight the complexity and invasiveness of CFLs, emphasizing the importance of adopting a multidisciplinary approach to manage this high-risk group of patients.

Following OE, various reconstructive techniques were used, including free flap (33.3%), endosseous implants (22.8%), and split-thickness skin grafting (17.2%). The most frequently used reconstructive material was the anterolateral thigh flap (26.6%), followed by the musculocutaneous flap (21.6%), and the radial forearm flap (14.5%). Several studies have proposed that free flaps and skin grafts can be employed individually or in combination to reconstruct the socket post-OE [2,33,49]. In terms of specific techniques, split-thickness skin grafting is favored due to its lower complication rates, while free flaps are typically reserved for more complex and extended OE, though not exclusively so [2,11,17,59]. A long-term study conducted by Baum et al. [15] has found that endosseous implants had a survival rate of 88.0% and were recommended for rehabilitation after OE. These findings underscore the importance of carefully selecting the appropriate reconstructive material based on the nature of the patient’s CFLs, the complexity and extent of exenteration, as well as the potential advantages offered by the material under consideration.

In our study, 168 patients (17.6%) experienced complications. The most frequent ones were sino-orbital or sino-nasal fistulas (22.6%), flap or graft failure (16.9%), and hyperostosis (13.0%). Several studies have documented complications following OE, encompassing sino-orbital fistulas, graft or flap failure, and surgical site infection or non-healing [5,7,53,60,61]. These complications can be ascribed to the anatomical site of the surgery, the nature of the reconstructive material employed, and the overall health status of the patient. One potential solution to mitigate surgical complications is to ensure the correct approach is taken with optimal visualization. This can lead to improved nasal symptoms and a reduction in related headaches, particularly in cases where a combined nasal approach is utilized [62,63].

Our study observed that a considerable portion of patients received radiotherapy (43.3%) and chemotherapy (4.9%), administered as primary, neoadjuvant, or adjuvant therapy. While the small size of the studies included in our review made it challenging to ascertain the potential benefit of adjuvant radiotherapy, numerous studies in the skull base literature have highlighted its effectiveness in enhancing local tumor control and diminishing tumor recurrence rates [64,65]. However, it is important to consider the potential complications of chemotherapy and radiotherapy and weigh the risks and benefits carefully. It is particularly noteworthy that radiotherapy has been significantly associated with the development of naso-orbital fistulas [11].

### 4.3. Clinical and Survival Outcomes

Over the course of the weighted mean follow-up period of 23.6 months, we observed a weighted mortality rate of 39.0% (95% CI: 28.0–50.0%). The majority of these deaths (73.8%) were due to disease-related complications. Our mortality rate is consistent with rates reported by other studies that have investigated similar pathologies. Rahman et al. [66] described a cohort of patients who underwent OE, with 43.8% being diagnosed with BCC. They reported an overall mortality rate of 38.0% over a 12-year period and concluded that after a 3-year period, a BCC patient’s risk of mortality increased by 30.4% compared to non-BCC patients. Similarly, Aryasit et al. [49] investigated a cohort where 35.9% of patients had SCC invading the orbit and reported a comparable mortality rate of 56.4%. Additionally, our review revealed a 5-year OS rate of 50.0%, with a median OS time of 61 months (95% CI: 46–83 months). These findings accord with earlier skull base studies of similar pathologies that were managed with various surgical approaches, which have demonstrated a 5-year OS rate ranging from 54.0% to 64.0% [56,67,68,69]. These results further substantiate our hypothesis that the unfavorable outcomes of OE are primarily due to the invasive nature of the included pathologies.

We found a local recurrence rate of 38.6%, a distant recurrence rate of 32.3%, and a regional recurrence rate of 6.7%, with tumors invading perineural and lymphovascular regions present in 17.4% and 5.0% of the patients, respectively. Similarly, many studies of craniofacial tumors in the literature corroborate these findings and reported local recurrence rates ranging from 8.5% to 36.0% [32,67]. However, our multivariable Cox proportional hazards model did not identify any significant predictors of OS, including tumor recurrence.

In the univariable analysis, we observed that the presence of positive surgical margins was associated with a shorter survival time (median: 24 months, 95% CI: 12–41 months) compared with negative surgical margins (median: 64 months, 95% CI: 36–NA months, *p* < 0.001). However, the association between the status of surgical margins and survival remains a topic of debate. While some studies have supported our findings and illustrated that positive surgical margins are associated with reduced survival, other studies failed to demonstrate a correlation between survival and tumor margin status [17,50,70,71,72]. One hypothesis is that micrometastases might already be present in cases of locally advanced tumors.

### 4.4. Limitations

The retrospective nature of our analysis poses limitations on our results. The articles included in our study were subject to significant selection biases and exhibited substantial heterogeneity in the methodology and the assessment of clinical outcomes. Additionally, the small sample sizes of most of the included articles reduced the statistical power of multiple endpoints.

## 5. Conclusions

OE is typically reserved for cases of advanced or recurrent CFLs. Despite the associated high rates of complications, recurrence, and mortality, our study suggests that it could be a valid option, provided that the management plan is meticulously tailored for each patient. To improve outcomes, rigorous postoperative monitoring and long-term follow-up are crucial. Such measures could potentially mitigate the devastating outcomes and poor prognosis associated with OE. Considering the profound psychological and social impacts on a patient’s self-image and quality of life, comprehensive rehabilitation and meticulous reconstruction following OE are strongly recommended.

## Figures and Tables

**Table 1:** Involved structures and clinical presentation.

Variable	*n* (%)
Involved structures	*n* = 989
Eyelids	160 (16.2%)
Orbit	142 (14.4%)
Conjunctiva	124 (12.5%)
Canthus	79 (8.0%)
Sinuses	59 (6.0%)
Face	41 (4.1%)
Lacrimal gland	37 (3.7%)
Choroid	23 (2.3%)
Nose	22 (2.2%)
Unspecified peri-oculus	19 (2.0%)
Periorbital skin	15 (1.5%)
Eye	14 (1.4%)
Cheek	10 (1.0%)
Brain	9 (0.9%)
Supraorbital/brow	8 (0.8%)
Lacrimal sac	7 (0.7%)
Ethmoid bone	6 (0.6%)
Lacrimal duct	4 (0.4%)
Maxilla	3 (0.3%)
Temporal skin	3 (0.3%)
Extraocular muscles	2 (0.2%)
Temple	2 (0.2%)
Alveoli	1 (0.1%)
Nasopharynx	1 (0.1%)
Ophthalmic nerve	1 (0.1%)
Optic nerve	1 (0.1%)
Parotid	1 (0.1%)
Periorbital fat	1 (0.1%)
Others	194 (19.6%)
Presenting signs and symptoms	*n* = 113 *
Disturbed vision	47 (22.5%)
Facial mass	37 (17.7%)
Conjunctival injection	31 (14.7%)
Eye pain	27 (13.0%)
Lesion discharge	25 (12.0%)
Epiphora	18 (8.6%)
Diplopia	16 (7.7%)
Orbital discomfort	8 (3.8%)

Data are reported as frequencies and percentages. * The total number of patients with symptoms does not sum up to the number of symptoms as many patients had multiple symptoms.

**Table 2:** Orbital exenteration and complications.

Variable	*n* (%)
Orbital exenteration extent of resection	*n* = 957
Total	302 (31.6%)
Extended	248 (26.0%)
Subtotal	87 (9.0%)
Unspecified	320 (33.4%)
Additional surgical interventions	*n* = 146
Maxillectomy	57 (39.0%)
Lymph node resection	36 (24.7%)
Ethmoidectomy	17 (11.6%)
Craniotomy	14 (9.6%)
Parotidectomy	5 (3.4%)
Craniofacial resection	4 (2.7%)
Enucleation	4 (2.7%)
Rhinectomy	3 (2.1%)
Tracheostomy	3 (2.1%)
Posterior orbit sparing	2 (1.4%)
Temporal bone resection	1 (0.7%)
Complications	*n* = 168 *
Sino-orbital/nasal fistula	40 (22.6%)
Flap/graft/implant failure (loss/necrosis/atrophy)	30 (16.9%)
Hyperostosis	23 (13.0%)
Wound dehiscence/non-healing	19 (10.7%)
CSF leak	15 (8.5%)
Surgical site infection	12 (6.7%)
Brain invasion	7 (4.0%)
Hematoma	6 (3.3%)
Socket hemorrhage	6 (3.3%)
Socket draining	3 (1.7%)
Transient facial weakness	3 (1.7%)
Myocardial infarction	2 (1.1%)
Stroke	2 (1.1%)
Pulmonary infection	1 (0.6%)
Delirium tremens	1 (0.6%)
Eyelid fistula	1 (0.6%)
Meningitis	1 (0.6%)
Orbital abscess	1 (0.6%)
Osteitis	1 (0.6%)
Permanent facial weakness	1 (0.6%)
Pulmonary edema	1 (0.6%)
Trigeminal neuralgia	1 (0.6%)

Data are reported as frequencies and percentages. CSF, cerebrospinal fluid. * The total number of patients with complications does not sum up to the number of complications as many patients had multiple and complications.

**Table 3:** Reconstructive techniques and materials.

Variable	*n* (%)
Reconstructive technique	*n* = 820
Free flap	273 (33.3%)
Endosseous implant	187 (22.8%)
Split-thickness skin graft	141 (17.2%)
Full-thickness skin graft	65 (8.0%)
Facial prosthesis	56 (6.8%)
Spontaneous granulation	54 (6.6%)
Orbital implant	24 (2.9%)
Surgical closure of the orbit	10 (1.2%)
Titanium plates	10 (1.2%)
Reconstructive material	*n* = 587
Anterolateral thigh flap	156 (26.6%)
Musculocutaneous flap	127 (21.6%)
Radial forearm flap	85 (14.5%)
Forehead flap	39 (6.6%)
Temporalis myofascial flap	39 (6.6%)
Lid flap	23 (3.9%)
Rectus abdominis flap	21 (3.6%)
Dermis-fat graft	19 (3.2%)
Latissimus dorsi flap	11 (1.9%)
Temporalis muscle flap	11 (1.9%)
Cheek rotation flap	10 (1.7%)
Facio-cervico-pectoral flap	9 (1.5%)
Pericranial flap	7 (1.2%)
Abdomen/sub-mammary skin	6 (1.0%)
Mustarde flap	4 (0.6%)
Cervicofacial flap	3 (0.5%)
Fibula osteocutaneous flap	2 (0.3%)
Lateral arm flap	2 (0.3%)
Scalp flap	2 (0.3%)
Buccal mucosa graft	1 (0.2%)
Deep inferior epigastric perforators flap	1 (0.2%)
Fasciocutaneous flap	1 (0.2%)
Galea frontalis flap	1 (0.2%)
Gastrocnemius flap	1 (0.2%)
Gracilis flap	1 (0.2%)
Parotid/left cheek skin	1 (0.2%)
Pectoralis major flap	1 (0.2%)
Temporoparietal fascia flap	1 (0.2%)
Thoracodorsal artery perforator flap	1 (0.2%)
Vastus lateralis flap	1 (0.2%)

Data are reported as frequencies and percentages.

**Table 4:** Non-surgical treatment: chemotherapy and radiotherapy.

Variable	*n* (%)
Chemotherapy	*n* = 47 ^†^
Neoadjuvant	16 (31.4%)
Adjuvant	35 (68.6%)
Radiotherapy	*n* = 414 ^†^
Primary ^a^	106 (25.0%)
Neoadjuvant	52 (12.3%)
Adjuvant	266 (62.7%)
Patients had both chemotherapy and radiotherapy.	*n* = 29

Data are reported as frequencies and percentages. ^a^ Primary refers to prior radiotherapy before orbital exenteration. ^†^ The total number of patients who received chemotherapy or radiotherapy does not sum up to the number of patients in adjuvant or neoadjuvant treatment groups as many patients had both treatments.

**Table 5:** Recurrence and clinical outcome.

Variable	*n* (%)/*n* (95% CI)
Recurrence	*n* = 210 *
Local	109 (38.6%)
Distant	91 (32.3%)
Regional	19 (6.7%)
Perineural invasion	49 (17.4%)
Lymphovascular invasion	14 (5.0%)
Surgical margins	*n* = 444
Positive	136 (30.6%)
Negative	308 (69.4%)
Status	*n* = 587
Alive	372 (63.4%)
Dead	215 (36.6%)
Death cause	*n* = 195
Disease complication	144 (73.8%)
Unrelated cause	51 (26.2%)
Overall median survival time, months	61 (95% CI: 46–83)
Overall survival rates	
5-year	50.0%
10-year	24.0%
Weighted mean follow-up time, months	23.6 (95% CI: 13.8–33.4)

Data are reported as weighted mean and 95% confidence interval (CI) or frequencies and percentages. * The total number of patients with recurrence does not sum up to the number of patients with each type of recurrence as many patients had multiple types of recurrences.

**Table 6:** Overall survival using Cox proportional hazards model of patient and treatment characteristics.

Predictor Variables	Overall Survival			
	Univariable Analysis		Multivariable Analysis	
	HR (95% CI)	*p*-Value	HR (95% CI)	*p*-Value
Age	1.01 (1.00–1.02)	0.04 *	1.03 (1.00–1.06)	0.09
Male (vs. female)	0.78 (0.54–1.11)	0.17	NA	NA
SCC (vs. BCC)	1.58 (0.92–2.72)	0.10	NA	NA
Melanoma (vs. BCC)	1.90 (1.09–3.32)	0.02 *	2.88 (0.70–11.9)	0.14
Total (vs. Subtotal) OE	1.25 (0.50–3.10)	0.63	NA	NA
Positive (vs. negative) margin	2.39 (1.48–3.86)	<0.001 *	2.04 (0.88–4.70)	0.10

Variables mentioned in parenthesis were used as “bases” when running the Cox proportional hazards model. * Association is significant at the 0.05 level (two-tailed). Bold text denotes statistical significance. HR, hazard ratio; CI, confidence interval; SCC, squamous cell carcinoma; BCC, basal cell carcinoma; OE, orbital exenteration; NA, not applicable.

## Supplementary Materials

The following are available online at: https://www.mdpi.com/article/10.3390/cancers15174285/s1, Figure S1: Funnel plot of (A) overall mortality rate of the cohort, (B) tumor recurrence rate based on the type of OE, and (C) mortality rate based on the type of OE. OE, orbital exenteration. Table S1: Risk of bias assessments for included studies. Table S2: Overview of clinical characteristics and outcomes of included studies.
