# Supplementary material for: Orbital Exenteration for Craniofacial Lesions: A Systematic Review and Meta-Analysis of Patient Characteristics and Survival Outcomes
Source: Cancers (Basel). 2023 Aug 27;15(17):4285. doi: 10.3390/cancers15174285 (PMC10487227; doi:10.3390/cancers15174285)
Supplement: Supplementary file 1 [file cancers-15-04285-s001.zip › cancers-2469356-supplementary.pdf]

Supplementary Materials: Orbital Exenteration for Craniofacial Lesions: A Systematic Review and Meta-Analysis of Patient Characteristics and Survival Outcomes

Jumanah Qedair, Ali S. Haider, Kishore Balasubramanian, Paolo Palmisciano, Taimur Hassan, Ataollah Shahbandi, Mohammadmahdi Sabahi, Abdurrahman F. Kharbat, Hussam Abou-Al-Shaar, Kenny Yu, Aaron A. Cohen-Gadol, Tarek Y. El Ahmadieh and Othman Bin-Alamer

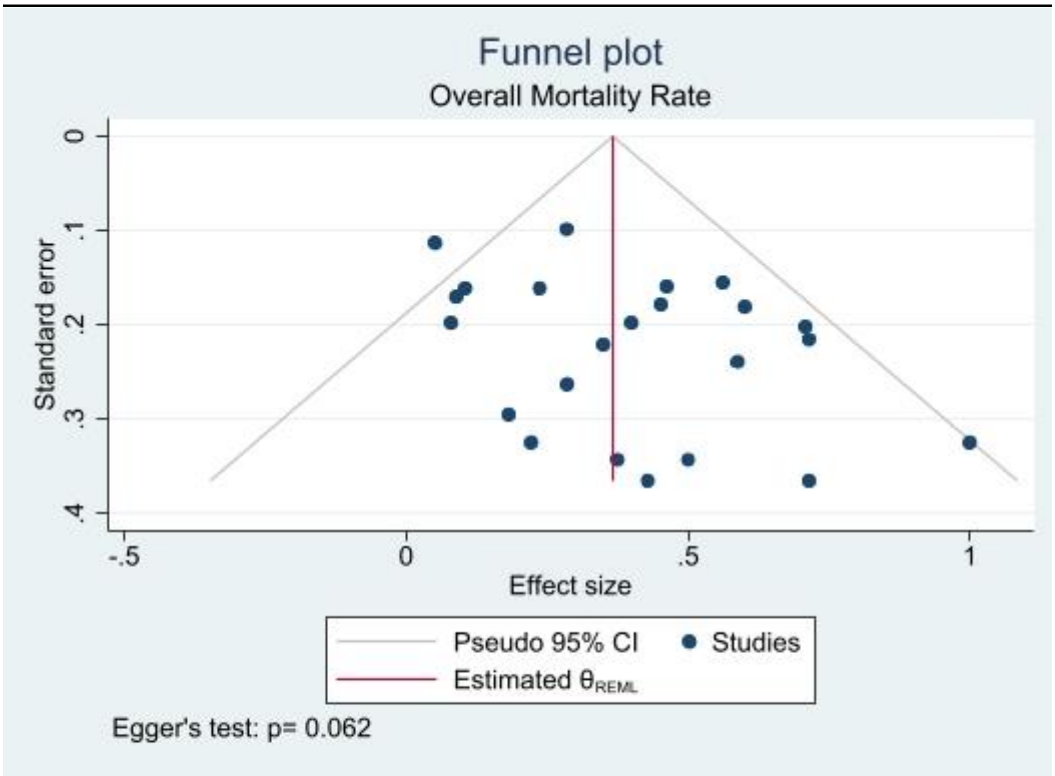

(**A**)

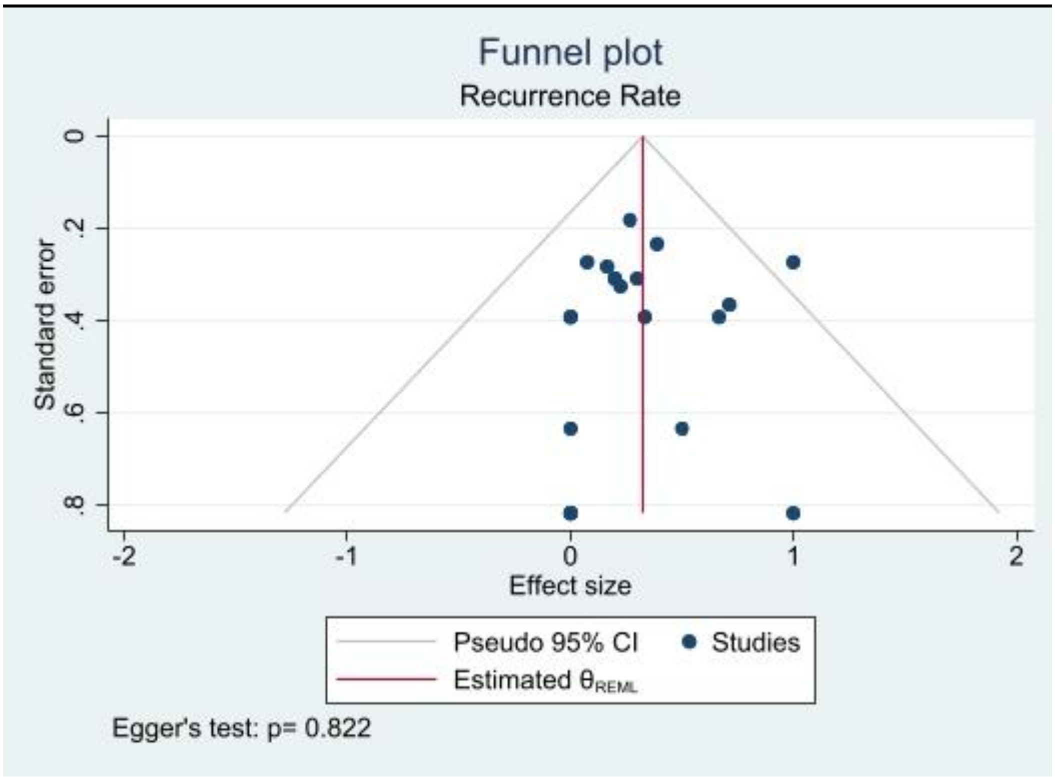

(**B**)

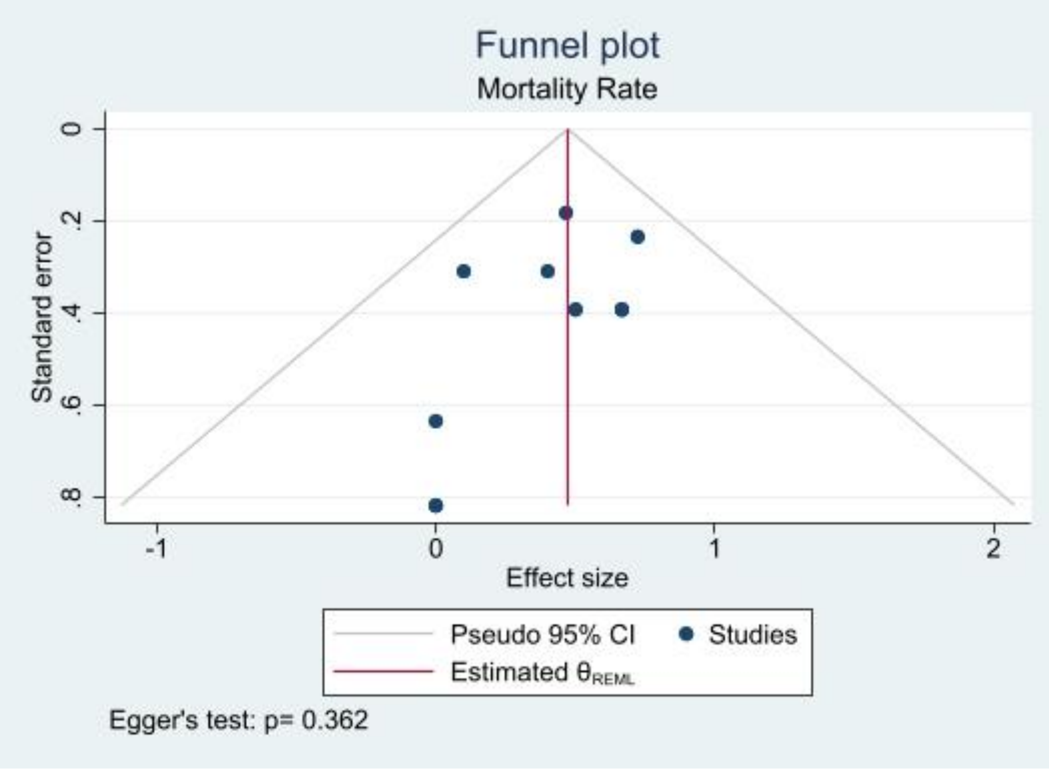

(**C**)

Figure S1. Funnel plot of **(A)** overall mortality rate of the cohort, **(B)** tumor recurrence rate based on the type of OE, and **(C)** mortality rate based on the type of OE. OE, orbital exenteration.

Table S1. Risk of bias assessments for included studies.

**JBI Checklist for Case Series – Criteria**
1. Were there clear criteria for inclusion in the case series?
2. Was the condition measured in a standard, reliable way for all participants included in the case series?
3. Were valid methods used for identification of the condition for all participants included in the case series?
4. Did the case series have consecutive inclusion of participants?
5. Did the case series have complete inclusion of participants?
6. Was there clear reporting of the demographics of the participants in the study?
7. Was there clear reporting of clinical information of the participants?
8. Were the outcomes or follow up results of cases clearly reported?
9. Was there clear reporting of the presenting site(s)/clinic(s) demographic information?
10. Was statistical analysis appropriate?
**Responses Options**: Yes, No, Unclear, Not Applicable (NA)
**Quality Rating**: Poor 0 – 3; Fair 4 – 7; Good 8 – 10

Study	1	2	3	4	5	6	7	8	9	10	Appraisal
Martel et al., 2020 [6]	Yes	Yes	Yes	Yes	Yes	Yes	Yes	Yes	Yes	Yes	10- Good
Nemet et al., 2007 [8]	Yes	Yes	Yes	Yes	Yes	Yes	Yes	Yes	Yes	Yes	10- Good
Hoffman et al., 2016 [9]	Yes	Yes	Yes	Yes	Yes	Yes	No	Yes	Yes	Yes	9- Good
Nassab et al., 2007 [23]	Yes	Yes	Yes	Yes	Yes	Yes	No	No	Yes	Yes	8- Good
Simon et al., 2005 [3]	Yes	Yes	Yes	Yes	Yes	Yes	Yes	Yes	Yes	Yes	10- Good
Tassone et al., 2017 [11]	Yes	Yes	Yes	Yes	Yes	Yes	No	Yes	Yes	Yes	9- Good
Taylor et al., 2006 [12]	Yes	Yes	Yes	Yes	Yes	Yes	Yes	Yes	Yes	Yes	10- Good
Zhang et al., 2018 [2]	Yes	Yes	Yes	Yes	No	Yes	Yes	No	Yes	Yes	8- Good
Baum et al., 2019 [15]	Yes	Yes	Yes	Yes	Yes	Yes	No	Yes	Yes	Yes	9- Good
Gill et al., 2017 [13]	Yes	Yes	Yes	Yes	Yes	Yes	No	No	Yes	Yes	8- Good
Kuo et al., 2011 [10]	Yes	Yes	Yes	Yes	No	Yes	Yes	Yes	Yes	Yes	9- Good
Lopez et al., 2013 [14]	Yes	Yes	Yes	Yes	Yes	Yes	Yes	Yes	Yes	Yes	10- Good
Qassemyar et al., 2014 [19]	Yes	Yes	Yes	Yes	Yes	Yes	No	Yes	Yes	Yes	9- Good
Cumming et al., 2019 [32]	Yes	Yes	Yes	Yes	Yes	No	No	Yes	Yes	Yes	8- Good
Gerring et al., 2017 [33]	Yes	Yes	Yes	Yes	Yes	Yes	No	Yes	Yes	Yes	9- Good
Catalano et al., 2001 [34]	Yes	Yes	Yes	Yes	Yes	Yes	No	Yes	Yes	Yes	9- Good
Coston et al., 1981 [43]	Yes	Yes	Yes	Yes	Yes	Yes	No	Yes	Yes	Yes	9- Good
Croce et al., 2008 [35]	Yes	Yes	Yes	Yes	Yes	Yes	No	Yes	Yes	Yes	9 – Good
Cuesta-Gil et al., 2004 [48]	Yes	Yes	Yes	Yes	Yes	Yes	No	Yes	Yes	Yes	9- Good
Elkhamary et al., 2017 [36]	Yes	Yes	Yes	Yes	Yes	Yes	No	Yes	Yes	Yes	9- Good
Elner et al., 1995 [46]	Yes	Yes	Yes	Yes	Yes	Yes	No	Yes	Yes	Yes	9- Good
Goldberg et al., 2003 [4]	Yes	Yes	Yes	Yes	Yes	No	Yes	Yes	Yes	Yes	9- Good

Karabekmez et al., 2014 [47]	Yes	Yes	Yes	Yes	Yes	Yes	No	Yes	Yes	Yes	9- Good
Lee et al., 2014 [37]	Yes	Yes	Yes	Yes	No	Yes	No	Yes	Yes	Yes	8- Good
Maheshwari et al., 2010 [38]	Yes	Yes	Yes	Yes	Yes	Yes	No	No	Yes	Yes	8- Good
Ogun et al., 2009 [39]	Yes	Yes	Yes	Yes	No	Yes	No	Yes	Yes	Yes	8- Good
Torroni et al., 2015 [40]	Yes	Yes	Yes	Yes	Yes	Yes	No	Yes	Yes	Yes	9- Good
Wang et al., 2021 [18]	Yes	Yes	Yes	Yes	Yes	Yes	Yes	Yes	Yes	Yes	10- Good
Esmaeli et al., 2006 [41]	Yes	Yes	Yes	Yes	Yes	Yes	No	Yes	Yes	Yes	9- Good
Lin et al., 2002 [44]	Yes	Yes	Yes	Yes	No	Yes	Yes	Yes	Yes	Yes	9- Good
Nagendran et al., 2016 [17]	Yes	Yes	Yes	Yes	Yes	Yes	Yes	Yes	Yes	Yes	10- Good
Naquin et al., 1954 [45]	Yes	Yes	Yes	Yes	No	Yes	No	Yes	Yes	Yes	8- Good
Rathbun et al., 1971 [42]	Yes	Yes	Yes	Yes	Yes	Yes	No	Yes	Yes	Yes	9- Good

Table S2. Overview of clinical characteristics and outcomes of included studies.

	Author, year	Level of evidence	Cohort size	Mean age ± SD	No. of males (%)	No. of SCC and BCC histopathology	OE type (T vs. S vs. E)†	Surgical margins (P vs. N)	Patient status
**1**	Martel et al., 2020 [6]	IV	25	63.2 ± 16.4	9 (36.0%)	SCC (4)<br>BCC (2)	T (22)<br>S (2)	P (4)<br>N (21)	Dead (10)<br>Alive (15)
**2**	Nemet et al., 2007 [8]	IV	38	69.9 (NS)	26 (68.4%)	SCC (15)<br>BCC (10)	T (26)<br>E (4)	P (14)<br>N (24)	Dead (9)<br>Alive (29)
**3**	Hoffman et al., 2016 [9]	IV	31	65 ± 15	24 (77.0%)	SCC (16)<br>BCC (8)	T (30)	-	Dead (14)<br>Alive (17)
**4**	Nassab et al., 2007 [23]	IV	32	68 (NS)	20 (62.5%)	SCC (3)<br>BCC (17)	-	-	-
**5**	Simon et al., 2005 [3]	IV	34	67 ± 14	10 (29.4%)	SCC (9)<br>BCC (6)<br>Mixed SCC/BCC (1)	T (14)<br>S (7)<br>E (7)	P (11)<br>N (23)	Dead (3)<br>Alive (31)
**6**	Tassone et al., 2017 [11]	IV	77	64.1 (NS)	57 (74.0%)	SCC (31)<br>BCC (15)	T (33)<br>S (5)<br>E (39)	-	Dead (4)<br>Alive (71)
**7**	Taylor et al., 2006 [12]	IV	14	66 (NS)	7 (50.0%)	SCC (2)<br>BCC (0)	T (10)	P (2)<br>N (12)	Dead (4)<br>Alive (10)
**8**	Zhang et al., 2018 [2]	IV	102	67.5 ± 15	55 (54.0%)	SCC (36)<br>BCC (17)	T (55)<br>E (47)	P (21)<br>N (81)	Dead (29)<br>Alive (58)
**9**	Baum et al., 2019 [15]	IV	48	62 ± 17	24 (50.0%)	SCC (15)<br>BCC (7)	T (22)<br>S (26)	-	-
**10**	Gill et al., 2017 [13]	IV	70	63.6 (NS)	53 (76.0%)	SCC (19)<br>BCC (12)	E (70)	-	-
**11**	Kuo et al., 2011 [10]	IV	38	68 (NS)	25 (65.8%)	SCC (19)<br>BCC (12)	T (7)<br>E (31)	P (17)<br>N (21)	Dead (4)<br>Alive (29)
**12**	Lopez et al., 2013 [14]	IV	21	58 ± 15	15 (71.4%)	SCC (6)<br>BCC (3)	T (5)<br>E (16)	P (7)<br>N (14)	Dead (15)<br>Alive (6)
**13**	Qassemyar et al., 2014 [19]	IV	26	68 (NS)	16 (61.5%)	SCC (9)<br>BCC (11)	T (13)<br>S (1)<br>E (12)	-	-
**14**	Cumming et al., 2019 [32]	IV	35	-	23 (65.7%)	SCC (15)<br>BCC (6)	-	P (12)<br>N (23)	-
**15**	Gerring et al., 2017 [33]	IV	49	70.3 (NS)	38 (77.6%)	SCC (17)<br>BCC (22)	-	P (12)<br>N (37)	-
**16**	Catalano et al., 2001 [34]	IV	20	57.85 ± 12.8	8 (40.0%)	SCC (7)<br>BCC (0)	-	-	Dead (7)<br>Alive (13)
**17**	Coston et al., 1981 [43]	IV	11	61.6 ± 15.7	5 (45.5%)	SCC (2)<br>BCC (1)	T (1)<br>S (10)	-	Dead (2)<br>Alive (9)
**18**	Croce et al., 2008 [35]	IV	8	75.2 ± 5.5	6 (75.0%)	SCC (1)<br>BCC (4)	T (6)<br>S (2)	-	Dead (4)<br>Alive (4)
**19**	Cuesta-Gil et al., 2004 [48]	IV	9	72.3 ± 9.4	6 (66.7%)	SCC (3)<br>BCC (4)	E (9)	-	Dead (2)<br>Alive (7)
**20**	Elkhamary et al., 2017 [36]	IV	27	64.1 ± 16.3	17 (63.0%)	SCC (11)<br>BCC (8)	T (13)<br>S (2)<br>E (5)	-	-
**21**	Elner et al., 1995 [46]	IV	8	60 ± 8.7	5 (62.5%)	SCC (4)<br>BCC (0)	-	-	Dead (3)<br>Alive (5)
**22**	Goldberg et al., 2003 [4]	IV	25	63.2 ± 9.9	-	SCC (7)<br>BCC (2)	T (13)<br>S (10)	P (7)<br>N (18)	Dead (2)<br>Alive (NS)
**23**	Karabekmez et al., 2014 [47]	IV	9	77 ± 6.3	2 (22.2%)	SCC (9)<br>BCC (0)	-	P (6)<br>N (3)	Dead (9)<br>Alive (0)
**24**	Lee et al., 2014 [37]	IV	17	54.6 ± 16.3	11 (64.7%)	SCC (6)<br>BCC (2)	-	-	Dead (10)<br>Alive (6)
**25**	Maheshwari et al., 2010 [38]	IV	15	53.9 ± 16.1	9 (60.0%)	SCC (3)<br>BCC (3)	T (2)<br>S (13)	-	-
**26**	Ogun et al., 2009 [39]	IV	11	56.5 ± 14.4	6 (54.5%)	SCC (11)<br>BCC (0)	-	-	-
**27**	Torroni et al., 2015 [40]	IV	9	75.1 ± 7.7	2 (22.2%)	SCC (3)<br>BCC (4)	T (6)<br>S (2)	-	-

							E (1)		
**28**	Wang et al., 2021 [18]	IV	30	66.8 ± 16.3	10 (33.3%)	SCC (2)<br>BCC (1)	-	P (10)<br>N (20)	Dead (18)<br>Alive (12)
**29**	Esmaeli et al., 2006 [41]	IV	7	43.7 ± 6.5	3 (42.9%)	SCC (0)<br>BCC (0)	E (7)	-	Dead (5)<br>Alive (2)
**30**	Lin et al., 2002 [44]	IV	7	56 ± 10.4	4 (57.1%)	SCC (3)<br>BCC (2)	T (6)<br>S (1)	-	Dead (3)<br>Alive (4)
**31**	Nagendran et al., 2016 [17]	IV	24	66.3 ± 18.4	14 (58.3%)	SCC (7)<br>BCC (3)	T (18)<br>S (6)	P (13)<br>N (11)	Dead (17)<br>Alive (7)
**32**	Naquin et al., 1954 [45]	IV	39	55.7 ± 9.4	25 (64.1%)	SCC (3)<br>BCC (11)	-	-	Dead (18)<br>Alive (19)
**33**	Rathbun et al., 1971 [42]	IV	41	59.6 ± 16.7	23 (56.1%)	SCC (6)<br>BCC (14)	-	-	Dead (23)<br>Alive (18)

Data are presented as number (%) or mean ± SD. †Might not sum up to the total cohort size as some patients' type of OE was not specified in the studies. SCC, squamous cell carcinoma; BCC, basal cell carcinoma; OE, orbital exenteration; T, total OE; S, subtotal OE; E, extended OE; P, positive surgical margin; N, negative surgical margin; NS, not specified.
